# The Absorption of Odors by Milk

**Published:** 1894-12

**Authors:** 


### The Absorption of Odors by Milk.

Parville relates some interesting facts upon this subject. If a can of milk is placed near an open vessel containing turpentine, the smell of turpentine is soon communicated to the milk. The same result occurs as regards tobacco, paraffin, asafœtida, camphor, and many other strong-smelling substances. Milk should also be kept at a distance from every volatile substance, and milk which has stood in sick chambers should never be drunk. The power of milk to disguise the taste of drugs—as potassium iodide, opium, salicylate, etc.—is well known.—*Gaillard's Med. Journal.*

